# Effect of stand age on rhizosphere microbial community assembly of dominant shrubs during sandy desert vegetation restoration

**DOI:** 10.3389/fpls.2024.1473503

**Published:** 2024-11-07

**Authors:** Yunfei Li, Bingyao Wang, Yanli Wang, Wenqiang He, Xudong Wu, Xue Zhang, Xiaorong Teng, Lichao Liu, Haotian Yang

**Affiliations:** ^1^ Key Laboratory of Ecological Safety and Sustainable Development in Arid Lands, Northwest Institute of Eco-Environment and Resources, Chinese Academy of Sciences, Lanzhou, China; ^2^ Shapotou Desert Research and Experiment Station, Northwest Institute of Eco-Environment and Resources, Chinese Academy of Sciences, Lanzhou, China; ^3^ University of Chinese Academy of Sciences, Beijing, China; ^4^ College of Forestry, Gansu Agricultural University, Lanzhou, China; ^5^ Institute of Forestry and Grassland Ecology, Ningxia Academy of Agricultural and Forestry Sciences, Yinchuan, China

**Keywords:** microbial community, metagenome, microbial functional genes, plant-soil interaction, desert ecosystem restoration

## Abstract

The rhizosphere microbial community helps govern biogeochemical cycling and facilitates complex plant-soil feedback. Understanding the evolutionary dynamics of microbial community structure and functional genes during vegetation succession is crucial for quantifying and understanding ecosystem processes and functions in restored sandy deserts. In this study, the rhizosphere microbial community structure of 11–66-year-old dominant shrubs in a desert revegetation area was examined using shotgun metagenomic sequencing. The interactions between the microbial community structure, functional gene abundances, soil properties, and plant characteristics of different stand ages were comprehensively investigated. The abundance of unique species first increased before subsequently decreasing with stand age, with shared species accounting for only 47.33%–59.42% of the total operational taxonomic units (OTUs). Copiotrophs such as Actinobacteria and Proteobacteria were found to dominate the rhizosphere soil microbial community, with their relative abundance accounting for 75.28%–81.41% of the total OTUs. There was a gradual shift in dominant microbial functional genes being involved in cellular processes towards those involved in environmental information processing and metabolism as stand age increased. Additionally, temporal partitioning was observed in both the microbial co-occurrence network complexity and topological parameters within the rhizosphere soil. Redundancy analysis revealed that dissolved organic carbon was the primary determinant influencing shifts in microbial community structure. Understanding the evolution of microbial community structure and function contributes to identifying potential mechanisms associating the soil microbiome with dominant sand-fixing shrubs as well as understanding the rhizosphere microbiome assembly process. These results shed light on the role of the rhizosphere microbiome in biogeochemical cycling and other ecosystem functions following revegetation of temperate sandy deserts.

## 1 Introduction

Although they have low productivity, dryland systems prominently influence global biogeochemical cycles and climate change, as they constitute 41% of the terrestrial land area (Safriel and Adeel, 2005). However, approximately 10%–20% of the world’s drylands are subject to desertification, soil degradation, and vegetation decline from anthropogenic disturbances and climate change (Safriel and Adeel, 2005). Vegetation restoration is an important practice for the containment and reversal of desertification, as well as an effective approach for rehabilitating degraded land (Zonn et al., 2017). Since the 1950s, China has made enormous efforts in ecological restoration and reconstruction, effectively combating deserts and reconstructing desert ecosystems through the installation of sand barriers and the planting of xerophytic shrubs (Korkanç, 2014; Gellie et al., 2017; Hu et al., 2021). This practice significantly impacts ecosystem health and global biochemical cycles due to its significant influences on ground cover (Hu et al., 2021; Scotton and Andreatta, 2021), soil hydrothermal conditions (Zhang et al., 2013; Li et al., 2022a), soil properties (Korkanç, 2014; Hu et al., 2021), and microbial community structure (Gellie et al., 2017; Huang et al., 2022).

Soil microorganisms play a pivotal role in fundamental ecosystem functions, serving as crucial players and mediators in soil biogeochemical cycles such as C sequestration and N retention (Baldrian et al., 2012; Sun and Badgley, 2019). Microbial communities are regulated by varying external factors such as available resources, plant inputs, plant age, environmental stresses, and biological interactions (Zumsteg et al., 2012; Knelman et al., 2014; Salles et al., 2017; Karliński et al., 2020; Qiao et al., 2024). Soil microbial communities undergo shifts along ecosystems age after restoration, mirroring changes in ecological conditions and exerting influence on the restoration of ecosystem functions (Sun et al., 2017; Sun and Badgley, 2019). However, many studies have focused only on describing alterations in microbial taxonomy (Zhao et al., 2018). Investigating changes in functional gene abundance involved in various ecological processes in soil microbial communities as restoration progresses provides a direct understanding of how revegetation affects ecosystem function (Sun and Badgley, 2019). Several studies have confirmed substantial shifts in the abundance of C- and N-cycle functional genes as restoration progresses (Brankatschk et al., 2011; Zeng et al., 2016; Salles et al., 2017; Sun and Badgley, 2019). The abundance of key genes involved in the microbial N cycle such as *nifH*, *nirS*, and *nirK* reached their maximum values at the middle stage of the restoration chronosequence (35 years) (Salles et al., 2017). Additionally, the abundance of *nirS* and ammonia oxidation genes decreased in some studies (Brankatschk et al., 2011), while others observed an increase in abundance with stand age (Zeng et al., 2016). Microbial functional genes related to the N and CH_4_ cycles varied linearly with restoration chronosequence in one study (spanning 6–31 years) (Sun and Badgley, 2019). Considering that the rhizosphere soil is the most intense site of plant-microbe-soil interactions, it is still unclear how the abundance of functional genes in rhizosphere soil microbial communities evolve or what the primary driving factors during the recovery of desert ecosystems are (Pausch and Kuzyakov, 2018).

Rhizosphere microbial community structure, specific functional genes, and ecological functions are closely related to stand age (Liu et al., 2018a; Song et al., 2022). As stand age increases, plants regulate the availability of soil nutrient resources through root exudates, which affects the formation of rhizosphere microbial community and functional gene abundance (Song et al., 2022). The microbial community diversity and the abundance of N and S cycling functional genes increased with stand age of *Eucalyptus* (Qu et al., 2020). Soil bacterial communities also showed a positive response with increasing stand age of *Robinia pseudoacacia* (Liu et al., 2018). Bacterial diversity, community structure, and potential metabolic functions in the rhizosphere also changed with the growth of *Spartina alterniflora* and *Kandelia obovate* (Song et al., 2022). However, in the special environment of desert ecosystems, the specific alterations in plant rhizosphere microbial community structure and functional gene groups with shrub age, along with their driving factors, remain unclear. This information is important in better guiding and managing desert revegetation systems.

The rhizosphere is a narrow area within 1–3 mm from the plant root surface and is influenced by plant growth and metabolic activity (Wang et al., 2016; Pausch and Kuzyakov, 2018). It represents a hot zone of heightened microbial activity and a major location for intricate interactions among plants, soil, and microorganisms. Rhizosphere microorganisms play indispensable roles in ecological and metabolic processes, serving as vital constituents of the rhizosphere (Zhang et al., 2021; Zhang et al., 2022). Changes in rhizosphere microbial communities not only directly reflect variations in vegetation and soil properties but also have significant implications for the formation and succession of microbial communities in bulk soil (Korniłłowicz-Kowalska et al., 2022; Zhou et al., 2022). Investigating rhizosphere microbial communities and their functional genes can provide a more direct understanding of how revegetation of desert ecosystems affects microbial processes associated with ecosystem function (Sun and Badgley, 2019; Yu et al., 2024), playing a critical role in revealing the impacts of long-term vegetation restoration on microbial communities.

This study leveraged metagenomics to monitor the temporal changes in microbial community structure and function along a chronosequence of restoration stand age in a desert revegetation area, aiming to investigate how the age of dominant shrubs affects soil microbial community assembly and functional gene group abundance as well as reveal the effects of dominant shrub stand age on rhizosphere soil microbial network complexity and stability. It was hypothesized that soil microbial community composition would evolve from oligotrophic to copiotrophic groups as the soil environment becomes more complex with increasing stand age, creating an increased need for cooperative, rather than competitive, relationships. Additionally, increasing stand age was expected to increase microbial network complexity and stability, enhancing microbial processes associated with ecosystem function. The results of this study will help to improve our understanding of the evolution of soil microbial communities and functions during the restoration of shrub-dominated desert ecosystems, as well as deepen our understanding of plant-microbe-soil interactions.

## 2 Materials and methods

### 2.1 Study area

The study area was situated within the revegetation zone at Shapotou, which lies on the southeastern fringe of the Tengger Desert in China (37°32′ N, 105°02′ E). The climate is characterized by aridity, low rainfall, and windy conditions. The annual average temperature is 10°C; the mean annual precipitation is 186.2 mm, and the mean annual evapotranspiration is 2800 mm. The soil is classified as wind-borne sand (Yang et al., 2020). The natural landscape is blowing dunes with vegetation coverage < 1%. The depth of the groundwater table is about 80 m from the surface, which is unavailable even to deep-rooted plants; rainfall serves as the sole resource for plant growth and development.

In the 1950s, a combination of checkerboard and shrub plantation was practiced to inhibit the erosion of mobile sand and keep the Lanzhou-Baotou railway from being buried. After the checkerboard of straw sand barriers was erected, xerophytic shrubs (mainly *Caragana korshinskii* Kom., *Artemisia ordosica* Krasch. and *Hedysarum scoparum* Fisch.) were planted at the checkerboard barriers with 16 individuals per 100 m^2^. Subsequently, this pattern was replicated along the north-south direction in 1964, 1973, 1987, 1999, and 2011. Presently, a protection system of rainfed artificial sand-fixing vegetation that is 16 km long and 0.7–1.0 km wide has been created. Following the establishment of the protection system, the composition of the vegetation community shifted from a shrub-dominated to a combined shrub and herbaceous pattern (Li et al., 2016). Soil properties, soil carbon sequestration (Yang et al., 2014; Li et al., 2022b), and microbial community structure (Liu et al., 2018b) were also significantly improved.

### 2.2 Soil sampling preparation

The soil samples for this study were collected in July 2022 from four revegetation sites constructed in 1956, 1964, 1987, and 2011, and were 66, 58, 35, and 11 years old, respectively. The soil type, elevation, slope, and aspect were similar at each sampling site. Three sampling plots (10 × 10 m) more than 50 m apart from each other were established at each revegetation area. Five *C. korshinskii* were investigated randomly across each plot, recording plant height, crown size, and basal diameter. The rhizosphere soil of corresponding plants was collected; the topsoil (0–20 cm) was removed as it did not have roots, then transects were established around the plant. Fine roots (< 1 mm) and attached rhizosphere soil were selected from the transects with sterile forceps, and loose soil was removed. The soil that remained adhered to the roots was considered rhizosphere soil. The fine roots with adhering soil were kept cold in the field and preserved in an ice box and then transported to the laboratory. Subsequently, the rhizosphere soil and fine roots were carefully separated on a sterile operating table. A total of 15 subsamples were collected, and a mixed sample was obtained by thoroughly mixing an equal number of the subsamples. Three replicate samples were collected at each site using the same method. Each composite sample was divided into two parts: one part was stored at −80°C and another was air-dried at room temperature for subsequent analyses.

### 2.3 Determination of soil physicochemical properties

The levels of total C (TC) and dissolved organic C (DOC) in rhizosphere soil were assayed using a TOC analyzer (Vario II, Elementar, Germany). Total nitrogen (TN) contents were determined using an element analyzer (Vario MACRO cube, Elementar INC., Germany). Soil pH was detected with a pH meter (CRISON 20, Barcelona, Spain) in a 1:5 soil–water mixture.

### 2.4 Shotgun metagenome sequencing and sequence processing

DNA was extracted from rhizosphere soil samples using the CTAB method (Yi et al., 2018). The DNA degradation degree, potential contamination, and concentration were measured using Agilent 2100 (Agilent Technologies Co. Ltd., USA) to screen qualified samples for sequencing. Sequencing libraries were generated using an NEBNext UltraTM DNA Library Prep Kit for Illumina (NEB, USA) and index codes were added to attribute sequences to each sample. Clustering of index-coded samples was performed on a cBot Cluster Generation System (Clark et al., 2011). After cluster generation, library preparations were sequenced on an Illumina Novaseq 6000 platform and 2 × 150 bp paired-end reads were generated. Raw sequencing data were filtered with Trimmomatic to obtain high-quality clean reads (Bolger et al., 2014). The clean reads after quality control and de-host were matched against a database (Uniref90) using Humann2 software; the resulting genes were searched against the NCBI and KEGG databases using Kraken 2 (v2.0.7-beta) for taxonomic and functional annotations (Segata et al., 2011; Lu et al., 2017; Franzosa et al., 2018).

### 2.5 Statistical analyses

Changes in the plant traits, soil properties, and relative abundances of phylogenetic groups and functional genes (KEGG level2) between samples were analyzed using single-variable methods with R. LEfSe analysis identified significantly different taxa at each stand age, and the impact of these species was further evaluated and downscaled using linear discriminant analysis (LDA) (Kitani and Murakami, 2020; Gojevic et al., 2022). The Bray-Curtis distances between sample pairs were estimated from species composition and visualized by non-metric multidimensional scaling (NMDS) using the ‘metaMDS’ function in the “vegan” package for R. The dissimilarity of communities between rhizosphere soil samples was assessed by generating a Venn diagram of shared and unique OTUs at 97% similarity. Soil microbial dominant phyla were visualized as heatmaps using the R package ‘pheatmap’, and the relationship between the abundance of major microbial taxa and stand age was evaluated by linear regression analysis.

Co-occurrence networks were constructed based on Spearman’s rank correlation coefficients of OTU abundances. First, OTUs whose average relative abundance was less than 0.1% and whose occurrence frequency was less than 1/10 of the total sample size were removed. Then, a pairwise similarity matrix (based on Spearman’s coefficients) and *p* values were computed for OTU abundances using the “WGCNA” package in R. Network diagrams were visualized in Gephi. In addition, regression analysis was conducted on the network complexity attribute, the network topological indices including *n* (total number of nodes), *L* (total number of links), modularity (the degree to which a network is compartmentalized into different modules), average degree, and positive and negative correlation links. Redundancy analysis (RDA) was performed to link microbial community structures to plant characteristics and soil properties using the “vegan” package in R. Variation partition analysis (VPA) was conducted to evaluate the impact of plant characteristics and soil properties on soil microbial community structure using Canoco 5.0.

To estimate the impact of stand age on soil properties, plant characteristics, microbial community structure, functional gene abundance, and microbial community co-occurrence network complexity, a partial least squares path model (PLS-PM) was built and tested. Soil properties were defined as a proxy including TN, TC, C:N, DOC, and pH. Plant characteristics were defined as a proxy including height, crown size, and basal diameter. Network complexity was defined as a proxy including *n*, *L*, modularity, average degree, and positive and negative correlation links. The microbial community structure was represented by the first and second principal components of the NMDS based on the OTU level. The functional gene abundance was represented by the first and second principal components of the principal components analysis based on significant differences in the OTU levels among different stand ages. A complete PLS-PM model was first built based on existing theories and concepts that included all the available variables mentioned above. Following this, variables with low contributions were deleted. The goodness of fit of the PLS-PM model was assessed. The PLS-PM model was built using SmartPLS 3.0 (Ramayah et al., 2018).

## 3 Results

### 3.1 Characteristics of plants and soil

The plant height and crown size initially increased significantly along the chronosequence of restoration, reaching maximum values at 58 y at 199.33 cm and 6.22 m^2^, respectively; after this, it decreased with increasing stand age (*p* < 0.05; **Table 1**). The basal diameter and the levels of TC and TN increased significantly along the chronosequence, whereas pH and C: N ratio decreased significantly (*p* < 0.05; **Table 1**). With the increase of stand age, the content of DOC first decreased and reached a minimum value at 35 y at 172.90 mg kg^-1^, before then increasing. At 66 y, the basal diameter increased by 426.10% compared to that at 11 y, and the contents of TC and TN increased by 69.23% and 139.13%, respectively. In contrast, the pH and C:N ratio had decreased by 3.72% and 30.39%, respectively (**Table 1**).

**Table 1:** Characteristics of plant, and rhizosphere soil properties along a chronosequence of revegetation.

Properties		11y	35y	58y	66y
Plant	Height (cm)	145.00 ± 9.00b	167.00 ± 7.55ab	199.33 ± 23.80a	125.33 ± 20.50c
	Crown size (m^2^)	0.92 ± 0.23c	3.08 ± 0.51b	6.22 ± 0.62a	3.96 ± 0.63b
	Basal diameter (cm)	2.49 ± 0.34d	6.54 ± 0.66c	9.69 ± 0.65b	13.10 ± 0.56a
Rhizosphere soil	TC (%)	0.39 ± 0.03b	0.44 ± 0.01b	0.44 ± 0.02b	0.66 ± 0.04a
	DOC (mg/kg)	264.68 ± 2.88a	172.90 ± 1.92d	193.20 ± 4.55c	223.68 ± 11.51b
	TN (g/kg)	0.23 ± 0.05b	0.26 ± 0.02b	0.28 ± 0.04b	0.55 ± 0.07a
	pH	8.06 ± 0.04a	8.11 ± 0.02a	7.92 ± 0.02b	7.76 ± 0.05c
	C: N ratio	17.34 ± 2.07a	17.21 ± 0.64a	15.68 ± 0.85ab	12.07 ± 1.05b

Values are mean ± SE. The different lowercase letters indicate significant differences at *p* < 0.05 level between the different stand age. TC, soil total carbon; DOC, dissolved organic carbon; TN, total nitrogen.

### 3.2 Microbial community composition of the rhizosphere soil

**Figure 1:**
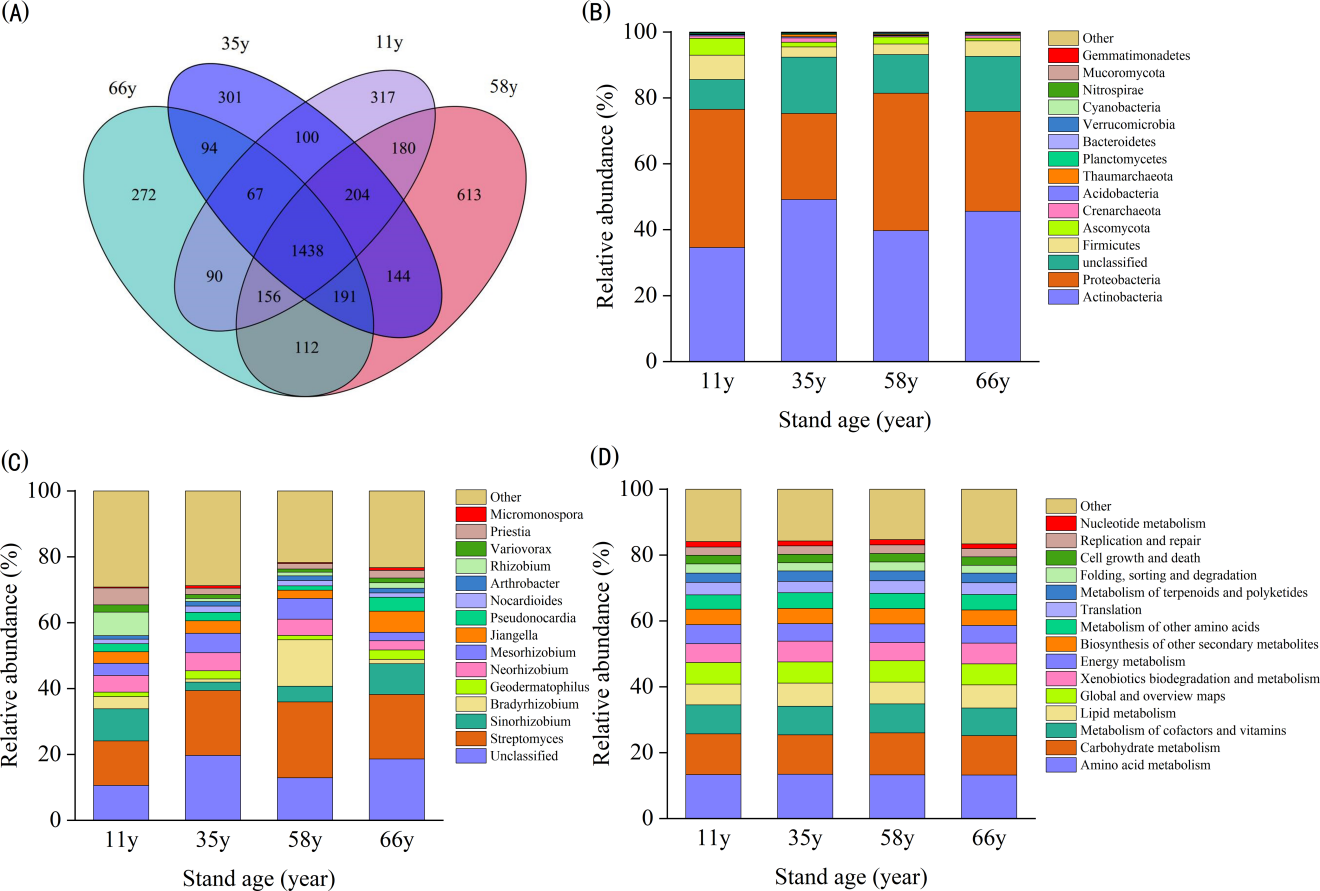
Rhizosphere soil microbial composition of different stand age: **(A)** Venn diagram of similarity and overlap of microbial composition of different stand age; **(B)** Bar diagram of top 15 dominant microbial composition at the phylum level across different stand age; **(C)** Bar diagram of top 15 dominant microbial composition at the genus level across different stand age; **(D)** Bar diagram of top 15 dominant microbial composition at the functional genes level (KEGG level2) across different stand age.

The microbial community composition in the rhizosphere soil of *C*. *korshinskii* showed significant differences between stand ages (*p* < 0.05; **Figure 1**). A total of 613 unique OTUs were obtained for the 58 y site, whereas unique OTUs for other sites ranged between 272–317 (**Figure 1A**). The rate of similar OTUs between different groups was 47.33%–59.42% (**Figure 1A**), indicating that the number of microbial communities affected by stand age was large.

Analysis of microbial community structures at the phylum, genus, and functional gene levels showed that the top 15 dominant species did not differ from 11- to 66-year-old samples, but did change in relative abundance (**Figures 1B–D**). At the phylum level, the main community members included Actinobacteria (34.59%–49.17%), Proteobacteria (26.11%–41.95%), Unclassified (9.04%–17.08%), Firmicutes (3.11%–7.37%), Ascomycota (0.82%–5.14%), Crenarchaeota (0.40%–1.31%), Acidobacteria (0.18%–0.37%), Thaumarchaeota (0.18%–0.75%), Planctomycetes (0.25%–0.29%), Bacteroidetes (0.11%–0.22%), and Verrucomicrobia (34.59%–49.17%). Proteobacteria, Firmicutes, and Ascomycota were more abundant in earlier samples, while Acidobacteria and Unclassified were more abundant in later samples (**Figure 1B**).

At the genus level, the main community members included *Unclassified* (10.57%–19.68%), *Streptomyces* (13.55%–22.99%), *Sinorhizobium* (2.54%–9.72%), *Bradyrhizobium* (0.96%–14.08%), *Geodermatophilus* (1.35%–2.84%), *Neorhizobium* (2.83%–5.52%), *Mesorhizobium* (2.53%–6.29%), *Jiangella* (2.52%–6.45%), *Pseudonocardia* (1.27%–4.21%), *Nocardioides* (1.34%–1.86%), *Arthrobacter* (1.06%–1.43%), *Rhizobium* (0.96%–7.14%), *Variovorax* (0.95%–2.23%), *Priestia* (1.65%–5.08%), and *Micromonospora* (0.32%–0.82%) (**Figure 1C**).

At the functional gene level, the percentage of relative abundance of the top 15 functional genes ranged between 83.37%–84.67%. The dominant gene groups in the community included amino acid metabolism (13.23%–13.46%), carbohydrate metabolism (11.96%–12.75%), metabolism of cofactors and vitamins (8.36%–8.81%), and lipid metabolism (6.35%–7.03%) (**Figure 1D**).

**Figure 2:**
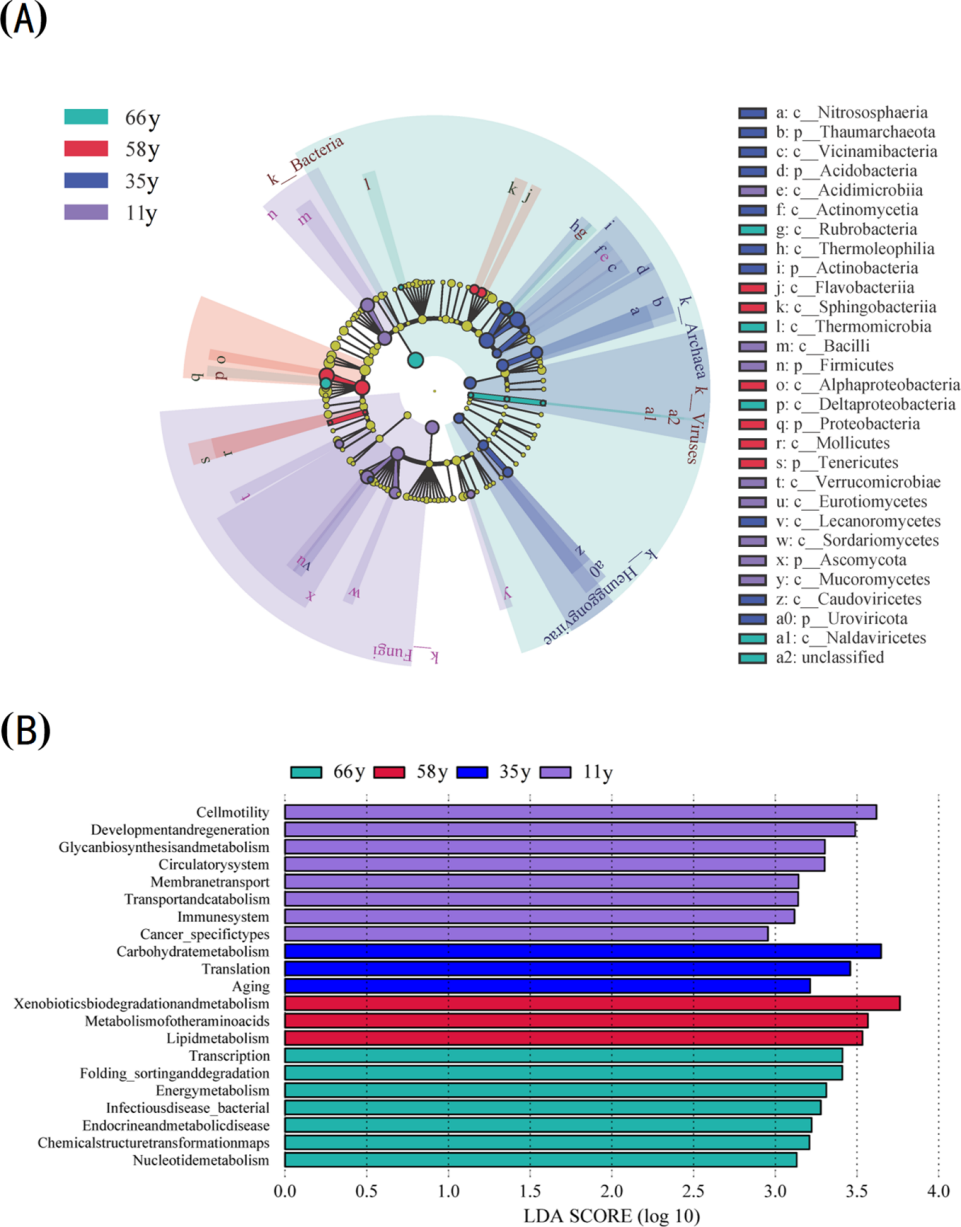
Analysis of biological markers with significant differences between different stand ages: **(A)** LEfSe cladogram of soil microbial among different stand ages; **(B)** Horizontal bar plot of the LDA scores calculated for features differentially abundant among stand ages. Alpha value for the factorial Kruskal-Wallis test among classes and for the pairwise Wilcoxon test between subclasses uses the default one. Threshold on the logarithmic LDA score for discriminative features is 2.0.

The LEfSe analysis indicated that there were significant differences in the relative abundance of eight phyla, 21 classes, and 21 functional gene groups between the four sites (LDA > 2) (**Figure 2**). More phyla and classes were enriched in the 11 and 66 y samples of rhizosphere soil (**Figure 2A**). At the class level, the main microorganisms enriched in 66 y soil included Thermomicrobia, Rubrobacteria, Deltaproteobacteria, and Naldaviricetes; those in 58 y soil included Flavobacteriia, Sphingobacteriia, Mollicutes, and Alphaproteobacteria; those in 35 y soil included Caudoviricetes, Vicinamibacteria, Actinomycetia, Lecanoromycetes, Thermoleophilia, and Nitrososphaeria; and those in 11 y soil included Verrucomicrobiae, Eurotiomycetes, Sordariomycetes, Bacilli, Acidimicrobiia, and Mucoromycetes.

At the functional gene level, the functional groups and numbers of microorganisms changed significantly along with stand age (**Figure 2B**). The numbers of functional groups with significant differences first decreased before increasing along with stand age. The functional genes with significant differences included glycan biosynthesis and metabolism, immune system, circulatory system, cell motility, membrane transport, cancer_specifictypes, transport and catabolism, and development and regeneration in 11 y samples, whereas transcription, folding_sorting and degradation, energy metabolism, chemical structure transformation maps, endocrine and metabolic disease, infectiousdisease_bacterial, and nucleotide metabolism were enriched in 66 y samples.

### 3.3 α-diversity of microbial communities in the rhizosphere soil

The *α*-diversity of rhizosphere soil microbial communities significantly differed with stand age (**Supplementary Table S2**). The richness estimate indices (Chao1, Shannon, Simpson’s) showed a turning point at 58 y. The Chao1 index gradually increased with stand age and reached the highest level at 58 y, before then decreasing with stand age. Shannon and Simpson’s indices decreased with the stand age and reached their minimum levels at 58 y, before then increasing.

### 3.4 β-diversity of microbial communities in the rhizosphere soil

**Figure 3:**
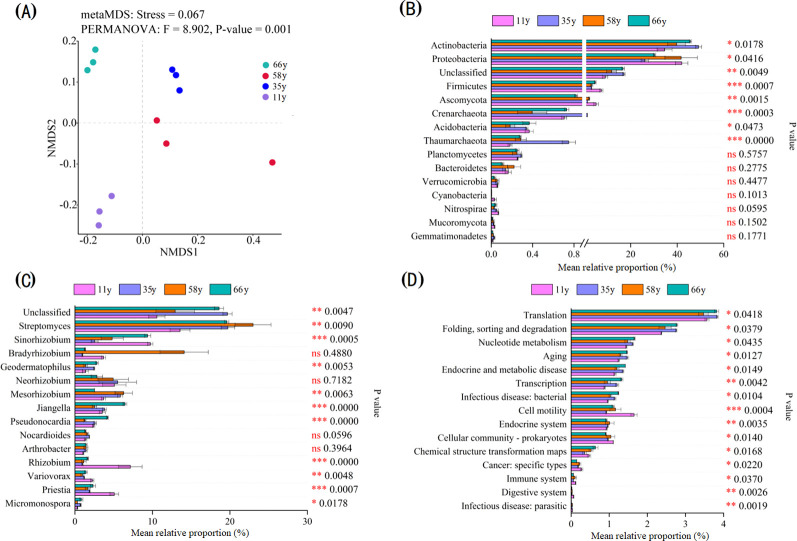
Microbial community beta diversity analysis and significance test for differences among stand ages: **(A)** The microbial community shift was visualized with NMDS based on species composition; **(B)** Significance test for differences among stand ages at major phylum (top 15 of relative abundance); **(C)** Significance test for differences among stand ages at major genus (top 15 of relative abundance); **(D)** Significance test for differences among stand ages at major functional gene (top 15 of relative abundance). The rightmost is the P value, * 0.01 < *p* ≤ 0.05, ** 0.001 < *p* ≤ 0.01, *** *p* ≤ 0.001.

The soil microbial community composition significantly differed between stand ages (*p* = 0.001). The patterns of *β*-diversity based upon species composition changed along with stand age from 11 to 66 y (**Figure 3A**). There were significant differences in rhizosphere soil microbial communities between stand ages at the phylum, genus, and functional gene levels (**Figures 3B, C**). There were significant differences in the relative abundances of microorganisms between stand ages in eight phyla (Actinobacteria, Proteobacteria, Unclassified, Firmicutes, Ascomycota, Crenarchaeota, Acidobacteria, and Thaumarchaeota; **Figure 3B**), 11 genera (*Unclassified*, *Streptomyces*, *Sinorhizobium*, *Geodermatophilus*, *Mesorhizobium*, *Jiangella*, *Pseudonocardia*, *Rhizobium*, *Variovorax*, *Priestia*, and *Micromonospora*; **Figure 3C**), and 16 dominant functional gene groups (**Figure 3D**). Actinobacteria, Thaumarchaeota, *Streptomyces*, and the folding, sorting and degradation genes group increased significantly in abundance with stand age, whereas Proteobacteria, Firmicutes, Ascomycota, *Rhizobium*, *Variovorax*, *Priestia*, and cell motility genes decreased.

### 3.5 Changes related to taxa and functional genes

**Figure 4:**
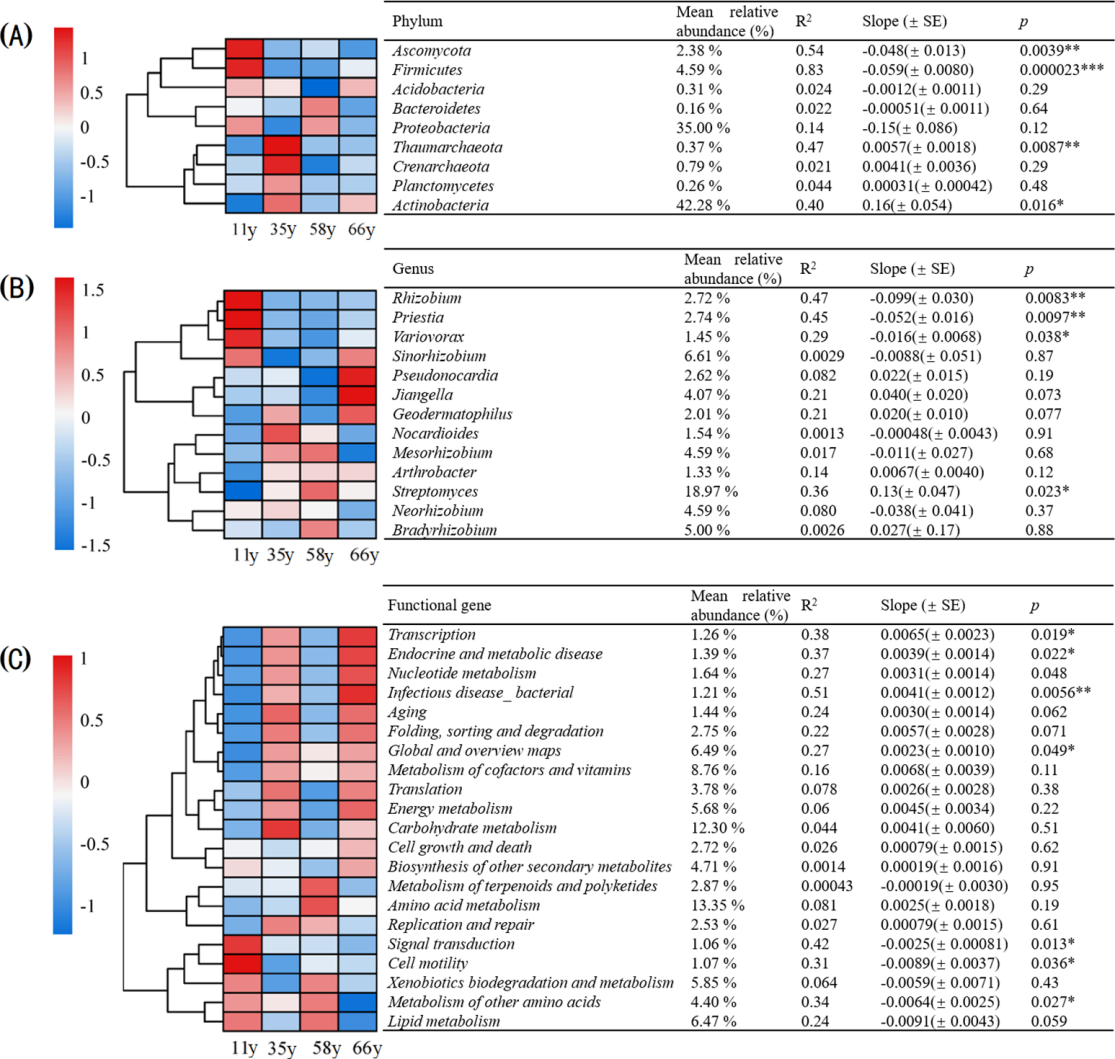
The variations of phyla, genus and functional genes over stand ages. The heatmap shows the age variations. The key shows the z-scores of the relative abundances. The relative abundance, variance explained (R^2^), regression slope and *P* value of the linear regression with stand age were shown in the table (*p < 0.05, **p < 0.01, ***p < 0.001): **(A)** The variations of major phyla over stand ages (relative abundance > 0.1%); **(B)** The variations of major genus over stand ages (relative abundance > 1%); **(C)** The variations of major functional genes over stand ages (relative abundance > 1%).

Multiple taxa with relative abundances > 0.1% varied across the chronosequence of stand ages at the phylum level (**Figure 4A**). Ascomycota and Firmicutes were more abundant at 11 y, whereas Acidobacteria were more abundant at 66 y. Linear regression analysis showed that the relative abundances of Ascomycota and Firmicutes significantly decreased with sample age from 5.14% and 7.37% at 11 y, respectively, to 0.82% and 4.70% at 66 y (*p* < 0.01, R^2^ = 0.54, 0.83); in contrast, the relative abundances of Thaumarchaeota and Actinobacteria significantly increased from 0.18% and 34.59% at 11 y to 0.28% and 45.63% at 66 y (*p* < 0.05, R^2^ = 0.47, 0.40) (**Figure 4A**).

There were also multiple taxa with relative abundances > 1% across the chronosequence of stand age that varied at the genus level (**Figure 4B**). *Rhizobium* and *Priestia* were more abundant at 11 y, whereas *Pseudonocardia* and *Jiangella* were more abundant at 66 y. The linear regression indicated that *Rhizobium*, *Priestia*, and *Variovorax* significantly decreased with age from 7.14%, 5.08%, and 2.23% at 11 y, respectively, to 1.63%, 2.33%, and 1.43% at 66 y (*p* < 0.05, R^2^ = 0.47, 0.45, 0.29); in contrast, the relative abundance of *Streptomyces* significantly increased from 13.55% at 11 y to 19.59% at 66 y (*p* < 0.05, R^2^ = 0.36) (**Figure 4B**).

The dominant functional genes with relative abundances > 1% were clustered based on the KEGG database at level 2. Among all categories, signal transduction and cell motility were more abundant at 11 y, whereas genes related transcription, energy metabolism, nucleotide metabolism, and endocrine and metabolic disease were dominant in later stages (**Figure 4C**). The relative abundance of transcription, endocrine and metabolic disease, infectious disease_bacterial, and global and overview maps increased significantly with stand age (*p* < 0.05), whereas the relative abundance of signal transduction, cell motility, and metabolism of other amino acids decreased significantly (*p* < 0.05) (**Figure 4C**).

### 3.6 Microbial community co-occurrence network complexity in the rhizosphere soil

**Figure 5:**
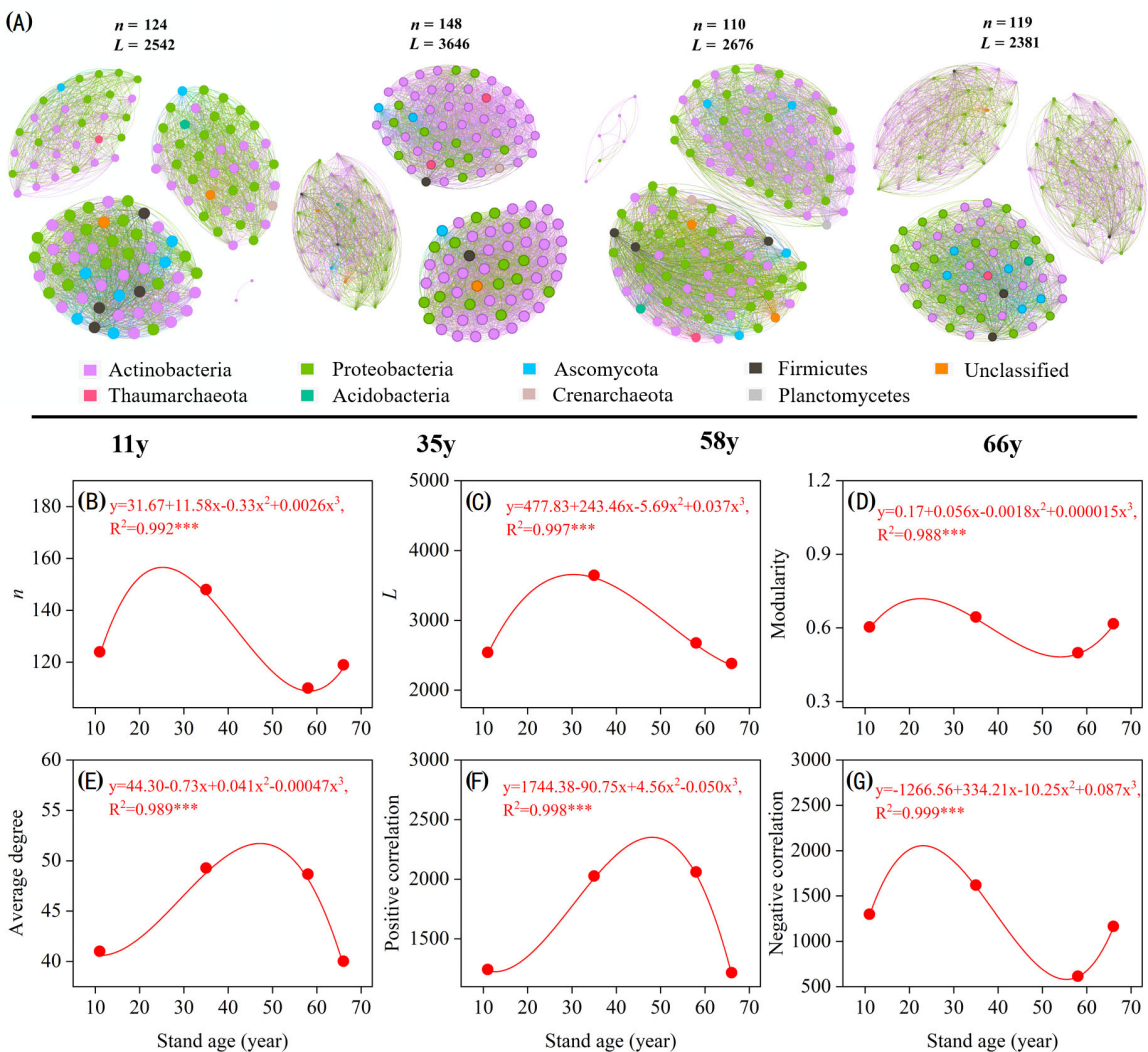
Rhizosphere microbial co-occurrence networks of with *Caragana korshinskii* different stand ages **(A)**. Connections indicate significant correlations (Screening conditions: Spearman’s ρ > 0.8, *p* < 0.001), the nodes are colored by phylum and represent an operational taxonomic unit (97% sequence identification threshold, OTU), the size of each node is proportional to the number of connections (degrees), and the thickness of each connection between two nodes (edge) is proportional to the values of Spearman’s correlation coefficient. Temporal changes in microbial community networks topology, including *n* **(B)**, *L* **(C)**, modularity **(D)**, average degree **(E)**, positivity correlation **(F)**, and negativity correlation **(G)**. The fitting formula and adjusted *R* ^2^ and *p* values from polynomial fitting are shown. * 0.01< *p* ≤ 0.05; ** 0.001 < *p* ≤ 0.01; ***p ≤ 0.001.

Stand age significantly altered rhizosphere soil microbial community complexity and stability (**Figure 5**). For microbial co-occurrence network complexity, the nodes, links, modularity, average degree, and positive and negative correlations all suggested “temporal partitioning” (**Figures 5A–G**). Co-occurrence network complexity increased with age during the first 35 years, reaching its highest at 35 y before experiencing a sudden decrease at approximately 58 y, then slowly increasing in the later stages (**Figures 5B–D**). The average degree and positive links sharply increased and reached their maximum at 48 y, before then decreasing with stand age (**Figures 5E, F**). The negative links in the microbial network first sharply increased with stand age and reached their highest level at approximately 21 y, decreasing to a lower level at approximately 55 y, and then increasing from 55 to 68 y (**Figure 5G**).

### 3.7 Influence of environmental variables on microbial community structures

**Figure 6:**
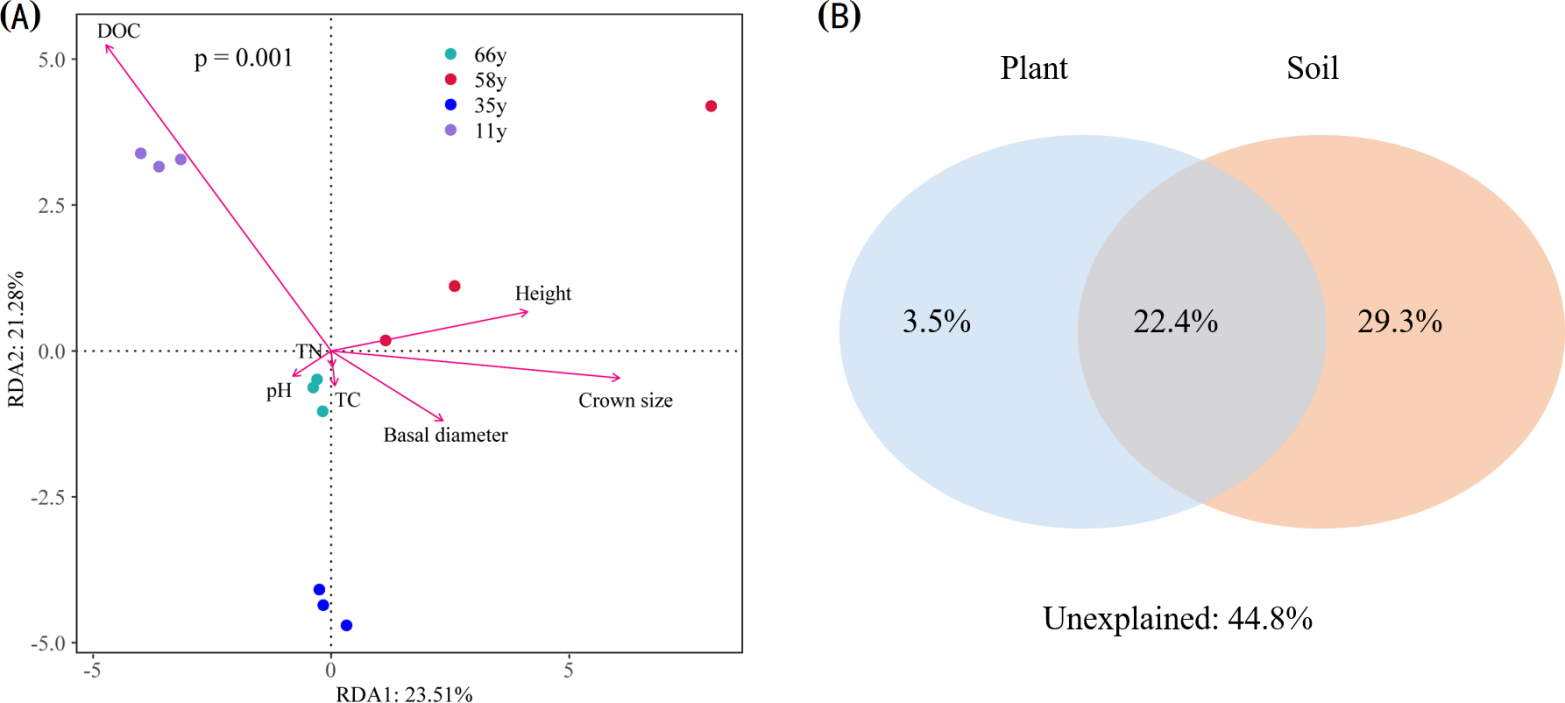
Redundancy analysis (RDA) **(A)** and variation partition analysis (VPA) **(B)** for the relationship between plant characteristics and soil properties and soil microbial communities across different stand ages.

The changes in microbial community structures were critically linked to the variations in plant traits and soil properties (**Figure 6A**). The first and second axes explained 23.51% and 21.28% of the variation in microbial community structure, respectively. The DOC content correlated most strongly with axis 1, with a correlation coefficient of 0.92. Permutation analyses showed that the DOC and crown size were the key factors that explained the variation in microbial community structures (*p* < 0.01) (**Figure 6A**). VPA results showed that the selected environmental variables explained 55.2% of the microbial community composition variation (**Figure 6B**). Soil properties independently explained the largest fraction of the variation (29.3%), plant variables independently explained 3.5% of the variation, and the interaction of soil and plants explained 22.4% of the variation (**Figure 6B**).

**Figure 7:**
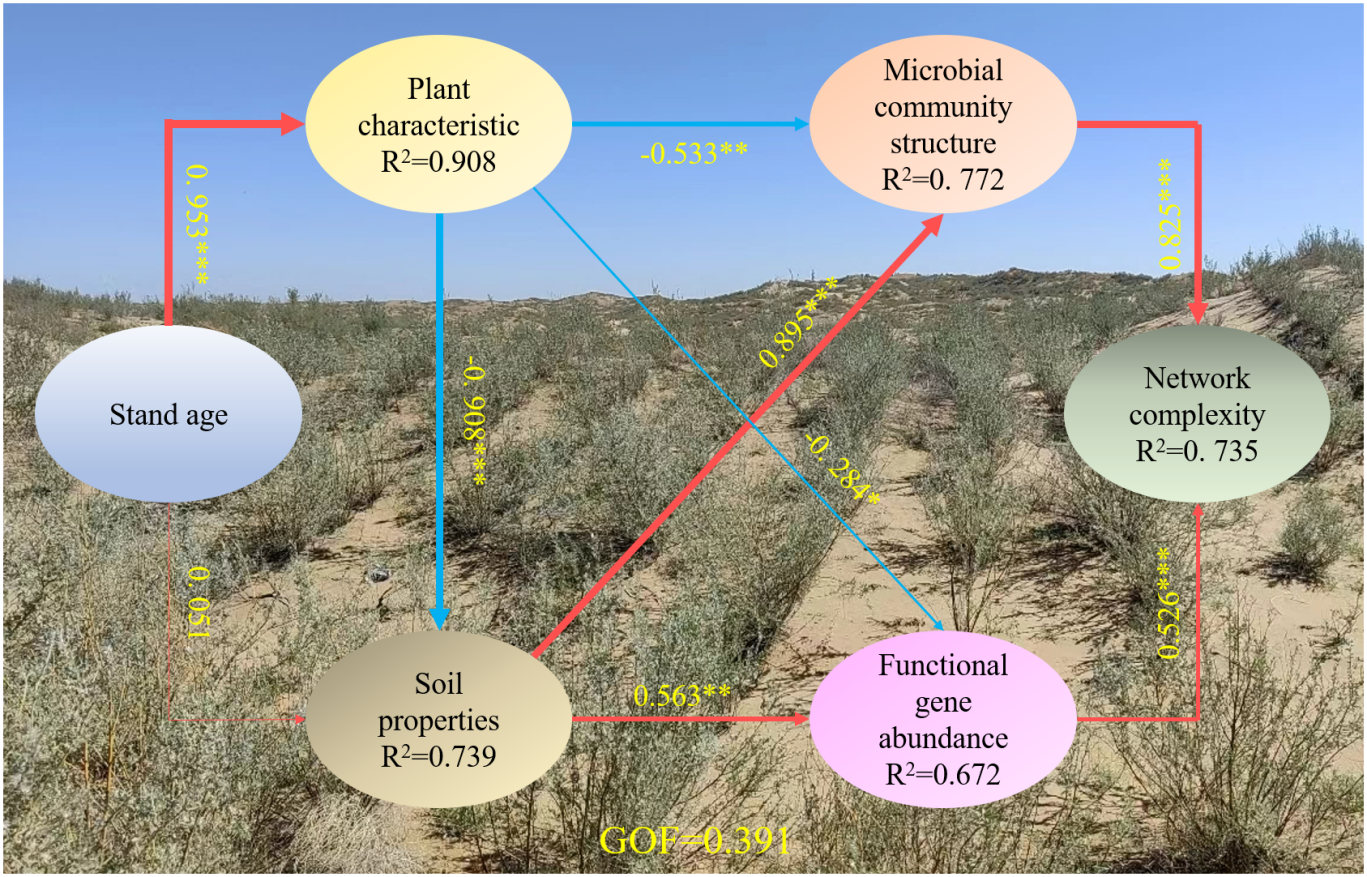
The direct and indirect effects of environmental factors on soil microbial community structure, functional gene and network complexity using partial least squares path models (PLS-PM). The width of the arrows is proportional to the strength of the causal relationship, and numbers are the correlation coefficients. Red solid lines indicate positive relationships and blue solid lines indicate negative relationships. R^2^ values represent the explained variance of each factor. The solid represent significant (***p < 0.001, **p < 0.01, *p < 0.05). The soil properties included DOC and pH; the plant characteristics included basial diameter and height; the microbial community structure included the first and second principal components of the NMDS based on the OTU level; the functional gene abundance included the first and second principal components of the PCA based on significant differences in the OTU levels among different stand ages; the network complexity included modularity and positivity correlation links.

Stand age directly drove changes in soil properties and plant characteristics, affecting changes in the microbial community structure, the relative abundance of functional genes, and the microbial community network complexity (**Figure 7**). Both microbial community structure and functional genes had significant positive effects on network complexity (*p* < 0.001). Plant characteristics had a significant negative impact on microbial community structure (*β* = –0.53, *p* < 0.01) and soil properties (*β* = –0.91, *p* < 0.001), whereas soil properties positively impacted microbial community structure (*β* = 0.90, *p* < 0.001). In addition, the positive effect of soil properties (*β* = 0.656, *p* < 0.01) was greater than the negative effect of plant characteristics (*β* = –0.28, *p* < 0.05) on functional gene abundance. There was a positive direct effect of stand age on plant characteristics (*β* = 0.95, *p* < 0.001), but the positive direct effect of stand age on soil properties was not significant (*β* = 0.01, *p* > 0.05) (**Figure 7**). The final model explained 73.9% of the variation in soil properties, 90.8% of the variation in plant characteristics, 77.2% of the variation in the microbial community structure, 67.2% of the variation in the relative abundance of functional genes, and 73.5% of the variation in the microbial community network complexity (**Figure 7**).

## 4 Discussion

Revegetation is one of the effective methods for rehabilitating degraded ecosystems, which has been widely adopted worldwide. In the desert ecosystem assessed in this study, there were significant changes in soil properties, nutrient effectiveness, hydrologic processes, plant diversity, and the structure and functional groups of soil microbial communities after revegetation. The quality and function of the environment gradually improved through the impact and regulation of plant-microbe-soil interactions. This study revealed the effects and mechanisms of dominant xerophytic shrub age on soil microbial community structure, functional groups, and network stability in temperate desert revegetation areas. It also sheds light on the ecological impacts of revegetation through soil microbial processes and provides new insights into our understanding of the relationship between deep-rooted plants and the environment.

### 4.1 Effects of stand age on rhizosphere microbial taxa

The microbial community composition of rhizosphere soil changed significantly with stand age at the phylum and genus level (**Figures 1**–**3**), which is consistent with previous studies (Sun and Badgley, 2019; Song et al., 2022). The strongest changes observed were the decrease in Ascomycota and Firmicutes and the increase in Actinobacteria, indicating a shift from oligotrophic to copiotrophic groups (Orwin et al., 2018; Yang et al., 2021; Liao et al., 2023). These shifts were closely related to soil property and plant traits. VPA analysis showed that the soil properties and plant characteristics selected in this study explained 55.2% of the variation in microbial community structure and that the effect of soil properties was much stronger than that of plants (**Figure 5B**). This implies that changes in plant traits induced by plant age indirectly impact soil microorganisms through variations in soil properties.

Actinobacteria and Proteobacteria were the dominant communities in rhizosphere soil. Actinobacteria play an important role in degrading organic matter such as polysaccharides, cellulose, starch, chitin, organic acids, proteins, and fats, as well as promoting plant growth (Hazarika and Thakur, 2020). Proteobacteria is one of the largest bacterial phyla, with significant metabolic multifunctionality, and plays a key role in C, N, S, and P cycling as well as that of metallic elements such as Fe and Mn (Wang et al., 2024). The abundance of Actinobacteria increased in a wave-like manner with stand age, while that of Proteobacteria showed an opposite trend (**Figure 1**). This can be attributed to changes in the soil environment, where improved soil nutrient conditions (such as increased SOC and TN) and the accumulation of plant secretions provided sufficient nutrients and a suitable environment for Actinobacteria (Dai et al., 2018).

Typically, the ratio of Proteobacteria/Acidobacteria represents the organic C quality of labile organic C pools, and its changes with stand age were consistent with the changes in DOC, correlating with most previous studies (Thomson et al., 2013). However, this is inconsistent with Sun et al. (2020) who found that the Proteobacteria/Acetobacteria ratio decreases with increasing TOC, which they attributed to the fact that the lower soil pH is more suitable for the growth of Acidobacteria. Proteobacteria and Bacteroidetes can metabolize complex organic molecules (Penn et al., 2014; Shao et al., 2021), and their reduced abundance with stand age implies a change in soil organic matter quality. The results suggest that soil C retention was better with increasing stand age; this was also a reasonable explanation for the gradual increase in TC content.

Rhizosphere soil microbial *α*- and *β*-diversity varied significantly with stand age (**Figure 3A**; **Supplementary Table S2**). The soil microbial Chao1 index first increased and then decreased with stand age, which was related to changes in plant traits and soil properties. The *β*-diversity increased with increasing stand age, implying that the heterogeneity of soil microbial communities was increased. This is due to the adaptation of the soil microbial community to the rhizosphere environment, especially the recovery of less resilient members. The variation in the abundance of dominant microorganisms as well as the lower number of unique OTUs at 66 y suggested that the cooperation among soil microorganisms increased with stand age and that they became more efficient in utilizing resources (Ren et al., 2016). The variation in rhizosphere soil microbial *β*-diversity could also be attributed to the consequence of habitat heterogeneity (Sun et al., 2020), similar to the findings of Sun et al. (2020) and verified by our RDA results (**Figure 5A**). As stand age increased, soil nutrient and organic matter content also increased significantly; these changes alleviate resource limitations for microbial growth, impacting the composition and diversity of the microbial community. Microbial network complexity initially increased and then decreased with stand age, matching the trend of plant traits (**Figure 5**; **Table 1**). Before trees reach maturity, the root secretions are dominated by lipids, sugars, and carbohydrates, which provide sufficient substrates for tree and microbial growth and promote the development of microbial networks; after the tree enters the mature stage, the secretions shift to defense-related substances, limiting the substrates available to microorganisms (Dayrell et al., 2018; Chen et al., 2023). Consequently, the microbial relationship network gradually becomes simpler and more stable.

Resource availability and microbial interactions are important environmental factors regulating changes in the soil microbiota during revegetation (Hoogmoed et al., 2016; Ren et al., 2016; Zhao et al., 2018). Our study showed that the spatial variation of soil microorganisms in the rhizosphere was associated with soil properties and plant characteristics (**Figure 5**). Soil pH was the key factor affecting the spatial and temporal distribution of bacteria, and a lower pH was more conducive to the colonization of acidic bacterial groups. The extractable C represents the available soil C pool, which is released by microorganisms in the form of respiration or incorporated into a more stable soil C pool through mineralization. The DOC gradually increased with stand age, providing more available substrates for microorganisms. The accumulation of rhizosphere soil C and N also resulted in a reduction in the C:N ratio (**Table 1**), suggesting that more labile substances were available for microbial processing and could increase plant productivity (Paul, 2007). Additionally, the changes in plant characteristics are also a major driver of microbial succession, since microbial community structure is related to variations in plant litter deposition and root exudates during revegetation (Zumsteg et al., 2012; Salles et al., 2017). A study on the Loess Plateau also obtained similar results; as the plant age increased, the bacterial community showed a positive response (Liu et al., 2018). As plant age increases, crown shading gradually increases, causing changes in microbial community structures (Song et al., 2022). The changes in plant characteristics affect soil properties and nutrients by modifying litter and root exudates; these alterations in soil nutrient resources lead to shifts in the microbial community composition, such as a gradual transition from oligotrophic to copiotrophic microbial groups. Ultimately, this influences the complexity and stability of the microbial community network (**Figure 7**).

### 4.2 Effects of stand age on rhizosphere microbial functional gene groups

Microorganisms are major players and key influencers of all biochemical processes (Gougoulias et al., 2014; Classen et al., 2015), and functional gene abundance reflects the microbial processes associated with ecosystem function (Sun and Badgley, 2019). In this study, the rhizosphere soil microbial functional groups changed significantly with stand age, being dominated by cellular processes (cell motility, development, and regeneration) in the early stages and by environmental information processing (transcription, chemical structure transformation maps) and metabolism (energy metabolism, endocrine and metabolic disease, nucleotide metabolism) functional groups in the later stages (**Figure 2**). This suggests faster biochemical cycling and higher resource effectiveness in rhizosphere soil, indicating that the interactions between plants and microbes were more intense in the later stages of restoration (66 y). Additionally, transcription, endocrine and metabolic diseases, infectious disease_bacterial, and global and overview maps groups increased linearly with shrub age (**Figure 4**); these changes were also driven directly or indirectly by changes in soil properties and plant characteristics caused by stand age (**Figure 7**). Previous studies have found that the younger trees secrete more metabolites related to nutrient acquisition, such as primary metabolites like carbohydrates and sugar. In contrast, older trees secrete more defensive metabolites in response to environmental changes (Dayrell et al., 2018; Chen et al., 2023). These changes in metabolite types reflect and result from alterations in microbial metabolic functions, which explains the results observed in this study.

Environmental factors that change with stand age also affect microbial function. A previous study found that *Rhizobiales* and *Cytophagales* were significantly positively correlated with the decomposition of plant residues because the degradation of plant residues (such as cellulose) is largely dependent on increased N availability achieved through N_2_ fixation (Tao et al., 2019). In the present study, these functional groups differed significantly among the different stand ages (**Figure 3C**), indicating that the ability to decompose organic matter changed with stand age and was associated with an increase of TN in rhizosphere soil. Song et al. (2022) found that the rhizosphere environment and biomass had a significant effect on the potential for N fixation with changing plant age. This was also confirmed in our study, where N-fixing rhizobia differed significantly in rhizosphere soils of different stand ages (**Figures 3**, **4**). Soil pH was also an important predictor of the abundance and diversity of C-degrading genes. Additionally, soil pH is considered an integrator factor for soil function; the gradual decrease in the abundance of Bacteroidetes in line with soil pH suggested that soil recalcitrant carbon will gradually accumulate, as this phylum is considered to specialize in the degradation of complex organic compounds (Fernandez-Gomez et al., 2013). This also implies that the ecological functions of the soil environment (such as carbon sequestration and enhanced nitrogen cycling potential) were progressively enhanced as the stand age increased.

### 4.3 Effects of stand age on the survival strategies of the rhizosphere microbial community

Soil nutrient resources shape the abundance of microbial communities and functional genes, and are also important factors in affecting the trophic phenotypes of soil microorganisms (Liao et al., 2023). Typical copiotrophic bacteria include Actinobacteria, Proteobacteria, and Bacteroidetes, whereas typical oligotrophic bacteria include Acidobacteria and Chloroflexi (Zechmeister-Boltenstern et al., 2015; Zhang et al., 2016). Here, the rhizosphere soil microbial community was dominated by copiotrophic bacteria (Actinobacteria, Proteobacteria, Bacteroidetes), although they varied in abundance between stand ages. This is consistent with previous research where microorganisms shifted from oligotrophic to copiotrophic groups with increasing restoration age (Zechmeister-Boltenstern et al., 2015; Sun and Badgley, 2019; Liao et al., 2023). This change is mainly related to variations in soil nutrient resources and properties, causing the competitive relationships within the soil microbial community to intensify and their utilization of nutrient resources to improve with increasing stand age.

Microbial functional genes are commonly considered microbial characteristics at the community level because even microorganisms of the same phylum also have different ecological functions due to physiological and developmental diversity (Chen et al., 2021a, b). The evolution of microbial functional genes in the process of vegetation restoration reflects a shift in microbial ecological strategies (Zechmeister-Boltenstern et al., 2015; Manoharan et al., 2017; Zheng et al., 2018). Generally, oligotrophic microorganisms are characterized by poor growth, greater affinity for substrates, preferential degradation of resistant C, and high metabolic rates (Starke et al., 2021); in contrast, microbial copiotrophs have vigorous growth, poor affinity for substrates, preferentially utilize labile C, and have high metabolic rates (Tan et al., 2019; Kelly et al., 2021). In this study, the predominance of copiotrophic groups in rhizosphere soil implies that soil microorganisms spent more energy on reproduction than on growth, metabolism, and improving competitiveness, which would favor the accumulation of resistant C pools (Tan et al., 2019; Kelly et al., 2021). This further suggests that changes in shrub age lead to alterations in plant characteristics and soil properties, driving changes in microbial community structure and functional genes. Consequently, this results in changes in the complexity of microbial networks. These findings indicate that ecological functions are gradually improved with increasing stand age under microbial regulation.

This study was conducted during the growing season, a period when plant life activities are at their peak and the dynamics of rhizosphere soil nutrients and microbial activities are most active (Huo et al., 2017). The microbial community structure and soil characteristics during this season best reflect the differences between forests of different ages. However, previous studies have reported that trees absorb nutrients from the soil even during the long winter dormancy period (Ueda et al., 2015; Zavišić and Polle, 2018), and can also release a certain amount of root exudates at the beginning of the dormancy season (Nakayama and Tateno, 2018). It is estimated that young trees release about 7.5% of their total below-ground C allocation as rhizosphere C flux during dormancy (Fahey et al., 2013). Additionally, soil microbial communities remain active at sub-zero temperatures and are capable of decomposing and mineralizing organic matter (Hishi et al., 2014; Isobe et al., 2018). During dormancy, plants could stimulate microbial growth and extracellular enzyme production to effectively absorb nutrients from the rhizosphere. The nutrients absorbed in winter are used for spring growth (Ueda et al., 2011; Nakayama and Tateno, 2022). Future study needs to assess winter rhizosphere processes to better understand forest soil nutrient cycling and plant growth. The results observed in this study may differ significantly from those in winter, potentially affecting our comprehensive understanding of plant-microbe interactions and plant growth strategies.

## 5 Conclusions

This study represents the first integration of soil microbial metagenomic information and driving factors to provide novel insights into alterations in both microbial community structure and functional genetics within the rhizosphere soil of dominant shrubs following vegetation restoration in desert ecosystems. Along four time series from 11 to 66 years after planting, significant changes were seen in the rhizosphere soil microbial community structure and functional genes that were closely related to plant characteristics and soil physicochemical properties. The restored desert ecosystem is more conducive to the accumulation of resistant organic matter in the soil due to the dominance of copiotrophic microorganisms such as Actinobacteria and Proteobacteria, which preferentially use labile substrates. In addition, the interactions between microorganisms become more robust in terms of cooperation and competition as stand age increases. This was attributed to a gradual shift of microbial functional genes from cellular processes to environmental information processing and metabolic processes. Additionally, changes in microbial community structure were primarily regulated by DOC, confirming the predominance of copiotrophic groups in the rhizosphere soil microbial community. Overall, this study establishes a foundation for comprehending the structure and functional genes of soil microbial communities, and their linkages with environmental changes in the rhizosphere of dominant shrubs during succession of sand-fixing vegetation. The results provide a novel perspective for understanding microbial processes in soil biogeochemical cycles in desert revegetation areas.

## Data Availability

The data presented in the study are deposited in the NCBI Sequence Read Archive (SRA) database, accession number PRJNA1178012, https://www.ncbi.nlm.nih.gov/bioproject/PRJNA1178012.
