# Supplementary material for: Effect of stand age on rhizosphere microbial community assembly of dominant shrubs during sandy desert vegetation restoration
Source: Front Plant Sci. 2024 Nov 7;15:1473503. doi: 10.3389/fpls.2024.1473503 (PMC11578715; doi:10.3389/fpls.2024.1473503)
Supplement: Supplementary file 1 [file Table1.docx]

Table S1 Variation explained of plant (height, crown size, basal diameter) and soil (TC, TN, DOC, pH) to the microbial community composition. A, plant; B, soil; C, interaction of A and B.

| Tested fraction | F | P |
| --- | --- | --- |
| A+B+C | 2.9 | 0.014 |
| A+C | 2.3 | 0.012 |
| B+C | 3.9 | 0.002 |

Table S2 Alpha diversity of microbial communities in the rhizosphere soil among stand ages.

|  | 11y | | 35y | | 58y | | 66y | |  |
| --- | --- | --- | --- | --- | --- | --- | --- | --- | --- |
| Chao1 | | 1685.06±38.70ab | | 1648.45±32.91ab | | 1867.41±112.03a | | 1576.99±28.52b | |
| Shannon | | 6.81±0.01a | | 6.84±0.07a | | 5.89±0.40b | | 6.41±0.02ab | |
| Simpson | | 0.97±0.00a | | 0.96±0.00a | | 0.92±0.03a | | 0.96±0.00a | |
